# A nine country study of the burden of non-severe nocturnal hypoglycaemic events on diabetes management and daily function

**DOI:** 10.1111/dom.12070

**Published:** 2013-02-25

**Authors:** M Brod, M Wolden, T Christensen, D M Bushnell

**Affiliations:** 1 The Brod GroupMill Valley, CA, USA; 2 Novo Nordisk A/SSoeborg, Denmark; 3 Health Research Associates, Inc.Mountlake Terrace, WA, USA

**Keywords:** diabetes complications, diabetes mellitus, glycaemic control

## Abstract

**Aims:**

The purpose of this study was to explore the burden and impact of non-severe nocturnal hypoglycaemic events (NSNHEs) on diabetes management, patient functioning and well-being in order to better understand the role that NSNHEs play in caring for persons with diabetes and facilitate optimal diabetes treatment management strategies.

**Methods:**

A 20-min survey assessing the impact of NSNHEs was administered to patients with self-reported diabetes age 18 or older via the Internet in nine countries (USA, UK, Germany, Canada, France, Italy, Spain, The Netherlands and Sweden) who experienced an NSNHE in the last month. Questions captured reasons for and length of the event, and impacts on diabetes management, daily function, sleep and well-being.

**Results:**

A total of 20 212 persons with Type 1 diabetes mellitus (T1DM) and Type 2 diabetes mellitus (T2DM) were screened of which 2108 respondents were eligible. Respondents initiated, on average, an additional 3.6 glucose monitoring tests, and did not resume usual functioning for an average of 3.4 hours after the NSNHE. Of the respondents using insulin, 15.8% decreased their insulin dose over an average of 3.6 days. NSNHEs also impacted sleep, with 10.4% not returning to sleep that night. Next day functioning was affected with 60.3% (n = 1273) feeling the need to take a nap and/or rest (with 65.5% of those actually taking a nap/rest) and 40.2% (n = 848) wanting to go to bed earlier than usual. A total of 21.4% were restricted in their driving the next day. These events also resulted in decreased well-being with 39.6% of respondents feeling ‘emotional low’ the following day.

**Conclusions:**

NSNHEs have serious consequences for patients. Greater attention to patient and physician education regarding the burden of NSNHEs and incorporation of corrective actions in treatment plans is needed to facilitate patients reaching optimal glycaemic control.

## Introduction

Non-severe hypoglycaemic events are not uncommon in both Type 1 diabetes mellitus (T1DM) and Type 2 diabetes mellitus (T2DM) and may occur in approximately one third of persons with diabetes with frequency of events as often as several times a week 1. Hypoglycaemic events represent a major challenge for patients, interfere with optimal long-term diabetes control, and contribute to excess morbidity and mortality 2–4. In addition, non-severe hypoglycaemia has been shown to negatively impact diabetes management, patient functioning and well-being, and result in work loss and reduced productivity 5–7. Furthermore, there is an economic burden for both patients and society as a result of increased blood glucose monitoring, health care resource utilization, reduced work productivity and patient out of pocket expenses 7.

Data from multiple studies indicate that non-severe hypoglycaemic events occur in approximately 24–60% of patients with diabetes 7–11 and can occur at any time of day or night while patients are at rest or engaged in activities. Both qualitative and quantitative research have found that non-severe nocturnal hypoglycaemic events (NSNHEs), occurring while patients are sleeping, create more fear and anxiety for patients than daytime events and have been found to result in greater work loss productivity than events that occur at work 1,7,12. NSNHEs may lead to medication non-adherence 1,5 and non-adherence is linked to among other deleterious effects issues with glycaemic control, hypoglycaemia and all-cause mortality 4,13,14. Furthermore, night-time events disrupt both sleep quality and quantity, resulting in impaired functioning and well-being the following day 5. Thus, previous research on NSNHEs have begun to suggest that these events are important barriers to achieving optimal glycaemic control and are not inconsequential contributors to increasing health care costs while reducing patient functioning and well-being.

The purpose of this study is to quantitatively explore, in greater depth than has previously been done, the burden and impact of these NSNHEs on diabetes management, patient functioning and well-being in order to better understand the role that NSNHEs play in caring for persons with diabetes and facilitate optimal diabetes treatment management strategies.

## Materials and Methods

### Survey Development and Conduct

A survey assessing the impact of NSNHEs was developed based on the literature, expert input and interviews with 78 persons with diabetes in nine focus groups in four countries (USA, UK, Germany and France) who recently had experienced an NSNHE. The survey items were developed based on a qualitative analysis of the expert input and the persons with diabetes interviews and cognitively debriefed and pilot tested in English in nine persons who met the same eligibility criteria as the focus groups. These steps were conducted to ensure content validity (relevant questions) and to ensure that the questions had face-validity with the respondents (e.g. no unfamiliar/strange words or concepts) 12. The final questionnaire was translated into all relevant languages using a forward and backward translation process. The survey was administered via a secure Internet server in the USA, UK, Germany, Canada, France, Italy, Spain, The Netherlands and Sweden.

NSNHEs were defined for the respondent as ‘night-time hypoglycaemic episodes that happened while you were sleeping and did not require medical attention (such as needing to call an ambulance, go to the emergency room/hospital) or did not require help from anyone else to manage the hypo. You knew that you were having this hypoglycaemic episode because you had symptoms like sweating and/or confusion or perhaps you experienced no symptoms, but noted the hypoglycaemic episode when measuring your blood sugar’. Respondents were asked questions regarding reasons for the event, length of time of the event, impact on productivity, daily functioning and well-being. The survey took approximately 20 min to complete and respondents were remunerated US $3–5 depending on country for completing the survey. The survey had several real-time validation steps (e.g. plausible min–max input values) and skip-patterns depending on the respondents reply. Prior to database-release, additional cross-checks were performed.

### Survey Sample

To be eligible to complete the survey, the respondent had to have a self-reported diagnosis of diabetes, be over 18 years of age, and be able to read the predominant language of the country they were living in. To minimize recall bias, respondents were required to have experienced at least one NSNHE in the last month. To ensure the generalizability of the results from the panel, the panel structure and recruitment used the following strategies: the panel used for the survey was multi-sourced; panellists were mainly recruited online via a wide range of permission e-mail recruitment, affiliate networks and website advertising, avoiding potential bias associated with single source recruitment methodology. Patients were recruited from several hundreds of websites as well as from face-to-face and telephone surveys where appropriate to include members who are not frequent online users. Additionally, the panel was used for research only; panellists were not exposed to third party advertising or direct marketing campaigns, nor were their personal data sold to third parties. The panel was also frequently refreshed to ensure that the panel was dynamic in nature and reflected any changes in the online population that might be occurring. Finally, the incentive was very low to help ensure that there was not undue incentive to participate in the panel.

The selection process used a sampling frame in a pre-existing panel of persons with self-reported T1DM or T2DM diabetes. All respondents went through a health care profiler (screening questions) to ensure that their diabetes had been diagnosed by a physician and that a relevant treatment was initiated. A stratified sampling procedure was employed using invitation selection criteria to account for disproportional response rates between stratification categories. Stratification variables were age (18–29 years, 30–49 years, 50–64 years and ≥65 years), diabetes type (T1DM and T2DM), gender and working status (working and non-working).

### Statistical Testing

Results by country are presented via frequencies or descriptives (means and s.d.) with differences explored using analysis of variance (anova) for continuous variables and Pearson chi-square for proportions. Statistical significance was tested between countries with the highest and lowest values. Analyses were conducted to characterize the last NSNHE, assess the impact of NSNHEs on diabetes management, assess the impact on functioning and well-being, and to compare nocturnal to daytime hypoglycaemic events. For the nocturnal-to-daytime comparison analyses, respondents assessed whether their night-time events were ‘more’, ‘about the same’ or ‘less’: (i) difficult to manage, (ii) severe, (iii) upsetting, (iv) physically impacting, (v) functionally impacting and (vi) frequent. For this article, we focused on the comparison of the extreme categories of ‘more’ and ‘less’. For questions with a 0–10 response scale where 0 is no impact and 10 being extremely impacted, scores were presented as means (with s.d.) and also categorized as none (0), mild (1–2), moderate (3–6) and severe (7–10).

## Results

### Sample Characteristics

A total of 20 212 respondents with self-reported diabetes were screened. Of these, 2108 respondents reported an NSNHE during the last 1 month and were also found eligible to complete the remaining survey (according to screening questions). Of these, 52.2% (n = 1100) reported working for pay. The overall recall period was short as 76.3% (n = 1609) reported having an NSNHE within the last 2 weeks.

The sample was equally divided between males and females (50%/50%) with a mean age of 49.9 years. The majority of the sample used insulin (74.2%) with the remainder on oral treatments only. The mean diabetes duration was 13.7 years. The majority of respondents reported experiencing an NSNHE at least several times a month (32.1%), 15.9% about once a week, 7.7% not daily but more than once a week and 0.9% daily. The remainder of the sample (43.3%) reported having NSNHEs once a month to very rarely (Table 1).

**Table 1:** Sample demographic characteristics

	Total	UK	Germany	USA	Canada	France	Italy	Spain	The Netherlands	Sweden	p-Value
Sample size with NSNHE within last month (N)	2108	305	279	501	200	193	138	242	169	81	
Age, Mean (s.d.)	49.9 (13.6)	48.4 (13.4)	49.0 (13.6)	53.7 (13.0)	54.5 (12.6)	45.9 (13.5)	44.6 (12.5)	42.2 (11.9)	55.3 (11.2)	54.7 (13.5)	0.000*
Range	20–89	20–81	20–85	20–89	22–83	20–83	20–76	20–77	22–81	23–81	
Gender; Female N (%)	1059 (50.2)	153 (50.2)	123 (44.1)	300 (59.9)	120 (60.0)	89 (46.1)	56 (40.6)	105 (43.4)	76 (45.0)	37 (45.7)	0.000†
Type of diabetes											0.000†
Type 1, N (%)	692 (32.8)	109 (35.7)	88 (31.5)	122 (24.4)	50 (25.0)	83 (43.0)	75 (54.3)	75 (31.0)	44 (26.0)	46 (56.9)	
Type 2, N (%)	1416 (67.2)	196 (64.3)	191 (68.5)	379 (75.6)	150 (75.0)	110 (57.0)	63 (45.7)	167 (69.0)	125 (74.0)	35 (43.1)	
Diabetes duration (years), Mean (s.d.)	13.7 (11.3)	13.6 (11.8)	11.8 (9.7)	14.8 (11.8)	14.4 (10.7)	13.7 (11.0)	14.2 (12.3)	10.2 (8.5)	14.0 (11.3)	21.4 (14.3)	0.000*
Range	0.1–75.6	0.4–57	0.1–49	0.3–60.7	0.3–48	0.3–50.3	0.1–75.6	0.3–49.6	1.4–70.3	1.6–58.3	
Number of medical complications, Mean (s.d.)	1.1 (1.2)	1.0 (1.2)	1.0 (1.0)	1.4 (1.3)	1.2 (1.2)	0.9 (1.1)	0.9 (1.0)	0.9 (1.0)	1.0 (1.0)	1.0 (1.3)	0.000*
Range	0–6	0–6	0–5	0–6	0–5	0–5	0–4	0–5	0–4	0–5	
Diabetes Treatment; N (%)											0.000†
Insulin	1565 (74.2)	210 (68.8)	226 (81.0)	351 (70.0)	139 (69.5)	156 (80.8)	106 (76.8)	164 (67.7)	135 (79.8)	78 (96.2)	
Orals Only	543 (25.7)	95 (31.1)	53 (18.9)	150 (29.9)	61 (30.5)	37 (19.1)	32 (23.1)	78 (32.2)	34 (20.1)	3 (3.70)	
Work for pay, N (%)											0.000†
Yes	1100 (52.2)	156 (51.1)	166 (59.5)	208 (41.5)	87 (43.5)	108 (56.0)	91 (65.9)	169 (69.8)	76 (45.0)	39 (48.1)	
No	1008 (47.8)	149 (48.9)	113 (40.5)	293 (58.5)	113 (56.5)	85 (44.0)	47 (34.1)	73 (30.2)	93 (55.0)	42 (51.9)	
Hours worked per week, Mean (s.d.)	36.8 (11.0)	33.7 (11.7)	38.6 (11.6)	36.5 (11.3)	35.7 (10.7)	38.2 (9.0)	37.0 (9.2)	40.2 (8.8)	32.3 (13.2)	35.3 (11.2)	0.000*
Frequency of NSNHE,‡ N (%)											
ALL (TYPE 1 and TYPE 2) (N = 2108)											0.000†
Daily	18 (0.9)	1 (0.3)	3 (1.1)	4 (0.8)	1 (0.5)	4 (2.1)	2 (1.4)	3 (1.2)	0 (0.0)	0 (0.0)	
Not daily, but more than once a week	163 (7.7)	15 (4.9)	13 (4.7)	59 (11.7)	14 (7.0)	20 (10.3)	11 (8.0)	19 (7.9)	10 (5.9)	2 (2.5)	
About once a week	336 (15.9)	61 (20.0)	48 (17.2)	72 (14.3)	17 (8.5)	35 (18.1)	23 (16.6)	37 (15.2)	28 (16.5)	15 (18.5)	
Several times a month	677 (32.1)	88 (28.8)	88 (31.5)	164 (32.7)	70 (35.0)	71 (36.7)	48 (34.7)	82 (33.8)	50 (29.5)	16 (19.7)	
Once a month	414 (19.6)	59 (19.3)	69 (24.7)	85 (16.9)	28 (14.0)	32 (16.5)	28 (20.2)	43 (17.7)	41 (24.2)	29 (35.8)	
Only a few times a year	401 (19.0)	68 (22.2)	49 (17.5)	83 (16.5)	58 (29.0)	25 (12.9)	23 (16.6)	47 (19.4)	36 (21.3)	12 (14.8)	
Very rarely	99 (4.7)	13 (4.3)	9 (3.2)	34 (6.8)	12 (6.0)	6 (3.1)	3 (2.2)	11 (4.5)	4 (2.4)	7 (8.6)	
TYPE 1 (n = 692)											0.002†
Daily	8 (1.2)	0 (0.0)	3 (3.4)	0 (0.0)	0 (0.0)	2 (2.4)	2 (2.7)	1 (1.3)	0 (0.0)	0 (0.0)	
Not daily, but more than once a week	52 (7.5)	5 (4.6)	0 (0.0)	20 (16.3)	2 (4.0)	6 (7.2)	8 (10.6)	5 (6.7)	4 (9.1)	2 (4.3)	
About once a week	134 (19.3)	27 (24.7)	18 (20.4)	22 (18.0)	5 (10)	18 (21.6)	14 (18.6)	9 (12.0)	9 (20.4)	12 (26.0)	
Several times a month	232 (33.5)	32 (29.3)	26 (29.5)	42 (34.4)	22 (44)	33 (39.7)	24 (32.0)	30 (40.0)	14 (31.8)	9 (19.5)	
Once a month	141 (20.3)	19 (17.4)	24 (27.2)	18 (14.7)	11 (22)	14 (16.8)	13 (17.3)	11 (14.6)	12 (27.2)	19 (41.3)	
Only a few times a year	115 (16.6)	23 (21.1)	17 (19.3)	17 (13.9)	10 (20)	9 (10.8)	12 (16.0)	18 (24.0)	5 (11.3)	4 (8.7)	
Very rarely	10 (1.4)	3 (2.8)	0 (0.0)	3 (2.4)	0 (0.0)	1 (1.2)	2 (2.7)	1 (1.3)	0 (0.0)	0 (0.0)	
TYPE 2 (n = 1416)											0.003†
Daily	10 (0.7)	1 (0.5)	0 (0.0)	4 (1.1)	1 (0.7)	2 (1.8)	0 (0.0)	2 (1.2)	0 (0.0)	0 (0.0)	
Not daily, but more than once a week	111 (7.8)	10 (5.1)	13 (6.8)	39 (10.2)	12 (8.0)	14 (12.7)	3 (4.8)	14 (8.4)	6 (4.8)	0 (0.0)	
About once a week	202 (14.2)	34 (17.3)	30 (15.7)	50 (13.1)	12 (8.0)	17 (15.4)	9 (14.2)	28 (16.7)	19 (15.2)	3 (8.6)	
Several times a month	445 (31.4)	56 (28.5)	62 (32.4)	122 (32.1)	48 (32.0)	38 (34.5)	24 (38.0)	52 (31.1)	36 (28.8)	7 (20.0)	
Once a month	273 (19.2)	40 (20.4)	45 (23.5)	67 (17.6)	17 (11.3)	18 (16.3)	15 (23.8)	32 (19.1)	29 (23.2)	10 (28.5)	
Only a few times a year	286 (20.1)	45 (22.9)	32 (16.7)	66 (17.4)	48 (32.0)	16 (14.5)	11 (17.4)	29 (17.3)	31 (24.8)	8 (22.8)	
Very rarely	89 (6.3)	10 (5.1)	9 (4.7)	31 (8.2)	12 (8.0)	5 (4.5)	1 (1.6)	10 (6.0)	4 (3.2)	7 (20.0)	

Ethnicity not available due to technical difficulty.

* Analysis of variance (anova).

† Chi square.

‡ About how often do you have night-time hypo episodes (while you are sleeping) that do not require medical attention (such as needing to call an ambulance or go to the emergency room/hospital) or do not require help from anyone else to manage the hypo episode?

### Characterization of the last NSNHE

The majority of events occurred during the hours of 2:00 a.m.–4:00 a.m. (41.3%) with another 33.2% occurring between midnight and 2:00 a.m.; 9.0% between 4:00 a.m. and 6:00 a.m.; and 5.3% before midnight. The majority of respondents (85.5%) were aware that they were experiencing their NSNHE because they had hypoglycaemic symptoms (self-identified or noticed by someone else) and approximately half of events (53.3%) were confirmed by blood glucose tests. Of note, 11.1% of respondents did not wake up during the event, but believed they had experienced a nocturnal hypoglycaemic event based on how they felt or their blood glucose reading upon awakening the next day.

It took an average of 7 min for respondents to realize they were having an NSNHE (after waking up) and another 6 min to do something about it (monitoring blood sugar, going to get something to eat or drink and then eating or drinking it) then 19 min for the acute hypoglycaemic symptoms to go away after eating or drinking something. Thus, the acute period of experiencing and managing the event took on average, approximately half an hour. The most common symptoms patients experienced during this time period were sweating (64.4%), shaking/tremors (42.0%), and restlessness, and tossing and turning in bed (35.7%); although the most bothersome symptoms reported were heart pounding or palpitations (11.7%), dizziness (9.6%) and sweating (8.7%). During the NSNHE, 6.9% (n = 145) of the respondents reported either tripping or falling and 31.0% (n = 45) injured themselves as a result of the fall. Of those that injured themselves, 26.6% (n = 12) required a visit to a doctor or health care professional.

**Figure 1:**
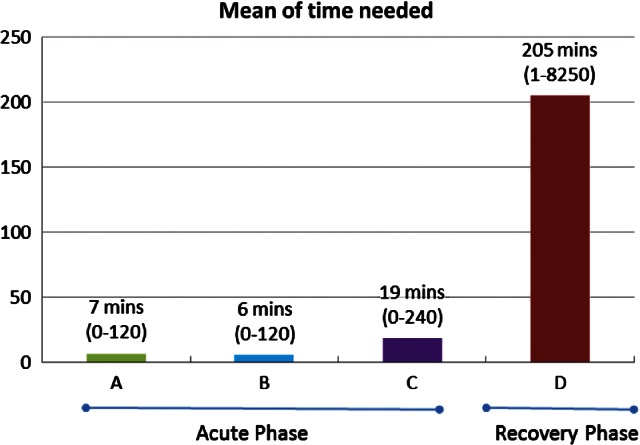
Mean time (minutes) required to deal with NSNHE, range in parenthesis. A = After you woke up that night, how long did it take to realize you were having a hypo before you did something about it? B = After you woke up that night, how long did it take after realization, to do something about the hypo including monitoring your blood sugar, going to get something to eat or drink and then eating or drinking it? C = After you woke up that night, how long did it take for all of your acute hypoglycaemic symptoms to go away after you had something to eat or drink? D = After you woke up that night, how long did it take before you felt like you were functioning again at your usual or normal level after you had something to eat or drink?

It took substantially more time on average (3.4 h or 205 min) before respondents felt like they were functioning again at their usual or normal levels (figure 1). Thus, the recovery phase for the event impacted the patient considerably longer than the acute phase.

### Impact of NSNHE on Diabetes Management

Compared to respondents' usual blood sugar monitoring practice, 3.6 (±6.6) extra tests were conducted, on average, in the week following the event. Of the respondents using insulin, 15.8% decreased their insulin dose after the NSNHE and the average decrease lasted 3.6 (±5.9) days (Table 2).

**Table 2:** Diabetes management after last NSNHE

	Total	UK	Germany	USA	Canada	France	Italy	Spain	The Netherlands	Sweden	p-Value
Sample size with NSNHE within last month (N)	2108	305	279	501	200	193	138	242	169	81	
How long ago did the NSNHE occur, N (%)											0.028*
2–3 days ago	402 (19.1)	64 (21.0)	35 (12.6)	123 (24.6)	35 (17.5)	35 (18.1)	33 (23.9)	37 (15.3)	26 (15.4)	14 (17.3)	
4–7 days ago	678 (32.2)	95 (31.1)	88 (31.6)	149 (29.7)	54 (27.0)	73 (37.8)	47 (34.1)	85 (35.1)	63 (37.3)	24 (29.6)	
1–2 weeks ago	529 (25.1)	73 (23.9)	76 (27.2)	125 (25.0)	58 (29.0)	35 (18.1)	33 (23.9)	67 (27.7)	43 (25.4)	19 (23.5)	
2–3 weeks ago	279 (13.2)	38 (12.5)	45 (16.1)	55 (11.0)	32 (16.0)	23 (11.9)	16 (11.6)	35 (14.5)	22 (13.0)	13 (16.0)	
3–4 weeks ago	220 (10.4)	35 (11.5)	35 (12.5)	49 (9.7)	21 (10.5)	27 (14.0)	9 (6.5)	18 (7.4)	15 (8.9)	11 (13.6)	
Time the hypo happened, N (%)											0.000*
Before midnight	111 (5.3)	21 (6.9)	11 (3.9)	33 (6.6)	8 (4.0)	5 (2.6)	11 (8.0)	14 (5.8)	3 (1.8)	5 (6.2)	
Midnight to 2:00 a.m.	700 (33.2)	108 (35.4)	100 (35.8)	177 (35.3)	53 (26.5)	69 (35.7)	44 (31.8)	85 (35.1)	47 (27.8)	17 (20.9)	
2:00 a.m. to 4:00 a.m.	872 (41.3)	114 (37.3)	104 (37.2)	187 (37.3)	101 (50.5)	81 (41.9)	65 (47.1)	84 (34.7)	96 (56.8)	40 (49.3)	
4:00 a.m. to 6:00 a.m.	190 (9.0)	22 (7.2)	17 (6.1)	42 (8.4)	15 (7.5)	19 (9.8)	10 (7.2)	34 (14.0)	16 (9.5)	15 (18.5)	
Missing data	235 (11.1)	40 (13.1)	47 (16.8)	62 (12.3)	23 (11.5)	19 (9.8)	8 (5.8)	25 (10.3)	7 (4.1)	4 (4.9)	
Status when last NSNHE happened, N (%)											0.000*
Woke up by symptoms	1625 (77.0)	237 (77.7)	187 (67.0)	383 (76.4)	155 (77.5)	159 (82.3)	101 (73.1)	180 (74.3)	152 (89.9)	71 (87.6)	
Had symptoms, woke up by others in the house	132 (6.3)	17 (5.6)	22 (7.9)	21 (4.2)	9 (4.5)	8 (4.1)	20 (14.4)	24 (9.9)	6 (3.6)	5 (6.2)	
Had no symptom, did not wake up	235 (11.1)	40 (13.1)	47 (16.8)	62 (12.3)	23 (11.5)	19 (9.8)	8 (5.8)	25 (10.3)	7 (4.1)	4 (4.9)	
Wake up regularly to check blood glucose	116 (5.5)	11 (3.6)	23 (8.2)	35 (7.0)	13 (6.5)	7 (3.6)	9 (6.5)	13 (5.4)	4 (2.4)	1 (1.2)	
Last NSNHE was, N (%):											0.000*
Symptomatic, confirmed by blood glucose test	1125 (53.3)	151 (49.5)	110 (39.4)	265 (52.8)	120 (60.0)	118 (61.1)	81 (58.6)	132 (54.5)	103 (60.9)	45 (55.5)	
Symptomatic, not confirmed by blood glucose test	679 (32.2)	106 (34.7)	102 (36.5)	157 (31.3)	50 (25.0)	54 (27.9)	44 (31.8)	79 (32.6)	56 (33.1)	31 (38.2)	
No symptoms, but confirmed by blood glucose test or by the way I felt when woke up	304 (14.4)	48 (15.7)	67 (24.0)	79 (15.7)	30 (15.0)	21 (10.8)	13 (9.4)	31 (12.8)	10 (5.9)	5 (6.2)	
Used to recover from last NSNHE,† N (%)											
Glucose tablets	325 (17.3)	69 (26.0)	41 (17.6)	88 (20.0)	30 (16.9)	10 (5.7)	11 (8.5)	15 (6.9)	51 (31.4)	10 (12.9)	0.000*
Glucose gel	52 (2.8)	10 (3.8)	12 (5.2)	9 (2.1)	2 (1.1)	6 (3.4)	4 (3.1)	7 (3.2)	2 (1.2)	0 (0.0)	0.124*
Sugar packages, honey or concentrated sweet syrup	257 (13.7)	9 (3.4)	56 (24.1)	16 (3.6)	15 (8.5)	41 (23.5)	46 (35.3)	42 (19.3)	21 (12.9)	11 (14.2)	0.000*
Candy, sweets, cake or biscuit/cookie	592 (31.6)	96 (36.2)	85 (36.6)	147 (33.4)	43 (24.2)	58 (33.3)	45 (34.6)	78 (35.9)	22 (13.5)	18 (23.3)	0.000*
Nutritional or sweet drink (e.g. soda, juice, sweet tea or milk)	732 (39.0)	96 (36.2)	103 (44.3)	152 (34.6)	76 (42.9)	74 (42.5)	36 (27.6)	106 (48.8)	57 (35.1)	32 (41.5)	0.001*
Sandwich, light meal or snack	488 (26.0)	92 (34.7)	37 (15.9)	136 (30.9)	59 (33.3)	31 (17.8)	9 (6.9)	39 (17.9)	56 (34.5)	29 (37.6)	0.000*
Full meal	21 (1.1)	4 (1.5)	3 (1.3)	3 (0.7)	2 (1.1)	2 (1.1)	2 (1.5)	3 (1.4)	1 (0.6)	1 (1.3)	0.984*
Nothing	44 (2.3)	4 (1.5)	7 (3.0)	11 (2.5)	4 (2.3)	7 (4.0)	1 (0.8)	4 (1.8)	5 (3.1)	1 (1.3)	0.663*
Other	163 (8.7)	14 (5.3)	24 (10.3)	52 (11.8)	14 (7.9)	8 (4.6)	11 (8.5)	11 (5.1)	16 (9.87)	13 (16.8)	0.002*
Number of extra blood sugar tests after last NSNHE, Mean (s.d.)											
First day (day of NSNHE)	1.3 (1.5)	1.3 (1.4)	1.3 (1.3)	1.1 (1.3)	1.4 (1.6)	1.1 (1.4)	1.4 (1.2)	1.8 (1.9)	1.3 (1.4)	1.0 (1.2)	0.000‡
Second day	0.6 (1.2)	0.6 (1.1)	0.5 (1.0)	0.4 (0.9)	0.8 (1.4)	0.6 (1.1)	0.9 (1.1)	1.1 (1.6)	0.5 (1.1)	0.2 (0.7)	0.000‡
Third day	0.4 (1.0)	0.4 (1.0)	0.3 (0.9)	0.3 (0.8)	0.5 (1.2)	0.5 (1.0)	0.6 (1.0)	0.9 (1.6)	0.3 (0.9)	0.1 (0.7)	0.000‡
Fourth to seventh day	1.3 (3.6)	1.1 (3.3)	0.8 (2.9)	0.8 (2.7)	1.4 (4.0)	1.5 (3.9)	1.6 (3.3)	3.0 (5.5)	0.9 (2.6)	0.5 (2.4)	0.000‡
Total (7 days after NSNHE)	3.6 (6.6)	3.4 (6.1)	3.0 (5.5)	2.5 (5.0)	4.2 (7.5)	3.8 (6.8)	4.5 (6.1)	6.8 (10.0)	3.0 (5.3)	1.8 (4.2)	0.000‡
Contacted a health care professional after last NSNHE, N (%)											
Yes§	313 (14.8)	36 (11.8)	61 (21.8)	40 (8.0)	29 (14.5)	33 (17.0)	35 (25.3)	58 (23.9)	16 (9.5)	5 (6.2)	0.000*
Did your LAST NSNHE cause you to decrease your normal insulin dose?											0.190*
Yes, N (%)	334 (15.8)	50 (16.3)	46 (16.4)	75 (14.9)	33 (16.5)	35 (18.1)	29 (21.0)	23 (9.5)	23 (13.6)	20 (24.6)	
No, N (%)	1160 (55.0)	150 (49.1)	174 (62.3)	254 (50.6)	101 (50.5)	111 (57.5)	73 (52.8)	131 (54.1)	109 (64.4)	57 (70.3)	
Missing (%)	614 (29.1)	105 (34.4)	59 (21.1)	172 (34.3)	66 (33.0)	47 (24.3)	36 (26.0)	88 (36.3)	37 (21.8)	4 (4.9)	
Days decreased, Mean (s.d.)	3.6 (5.9)	2.4 (4.3)	2.7 (2.7)	2.7 (5.2)	4.8 (7.4)	3.1 (5.7)	5.5 (6.6)	4.1 (5.4)	7.0 (9.7)	2.5 (6.5)	0.058‡
*Falls and Trips*											
Did you trip and/or fall during this night-time hypo?											0.003*
Yes, N (%)	145 (6.9)	25 (8.2)	23 (8.2)	27 (5.4)	6 (3.0)	21 (10.8)	8 (5.8)	25 (10.3)	5 (3.0)	5 (6.2)	
No, N (%)	1728 (81.9)	240 (78.6)	209 (74.9)	412 (82.2)	171 (85.5)	153 (79.2)	122 (88.4)	192 (79.3)	157 (92.8)	72 (88.8)	
Missing (%)	235 (11.1)	40 (13.1)	47 (16.8)	62 (12.3)	23 (11.5)	19 (9.8)	8 (5.8)	25 (10.3)	7 (4.1)	4 (4.9)	
When you trip/fall, did you hurt yourself?											0.011*
Yes, N (%)	45 (31.0)	12 (48.0)	1 (4.3)	7 (25.9)	0 (0.0)	7 (33.3)	2 (25.0)	12 (48.0)	3 (60.0)	1 (20.0)	
No, N (%)	100 (68.9)	13 (52.0)	22 (95.6)	20 (74.0)	6 (100.0)	14 (66.6)	6 (75.0)	13 (52.0)	2 (40.0)	4 (80.0)	
Did this trip/fall require a visit to doctor or other health professional?											0.493*
Yes, N (%)	12 (26.6)	4 (33.3)	0 (0.0)	1 (14.2)	0 (0.0)	1 (14.2)	1 (50.0)	4 (33.3)	0 (0.0)	1 (100.0)	
No, N (%)	33 (73.3)	8 (66.6)	1 (100.0)	6 (85.7)	0 (0.0)	6 (85.7)	1 (50.0)	8 (66.6)	3 (100.0)	0 (0.0)	

* Chi square.

† These categories are mutually exclusive.

‡ Analysis of variance (anova).

§ Primary care doctor, hospital, diabetes clinic, or other health care professional.

**Figure 2:**
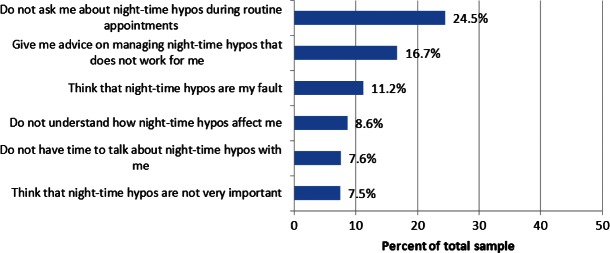
Health care provider interactions.

Of the respondents, 66.1% (n = 1394) discussed NSNHEs with their health care providers during regularly scheduled visits and most of these respondents, 94.8% (n = 1321), felt that they received helpful information about how to manage these events in terms of their diabetes management or the impact of the event on their functioning and well-being. However, a smaller percent reported that their health care providers blamed them for their hypoglycaemic events or did not understand how much these events impacted them (figure 2).

In addition, 14.8% (n = 313) of the total sample contacted a health care professional (either primary care physician, hospital, diabetes clinic or other health care worker) as a result of the event (e.g. for advice on medication, exercise or eating habits).

### Impact of Last NSNHE on Functioning and Well-being

#### Impact on Sleep

Respondents reported a moderate impact (mean 5.1 on 10-pt scale with 10 being prevented from going back to sleep) of the NSNHE on their sleep the night of the event. For those who woke up due to the NSNHE (88.9%, n = 1873), the average time it took people to fall back to sleep was 62.6 min and 10.4% (n = 194) never went back to sleep, remaining awake for the remainder of the night. A small number of respondents (3.8%, n = 81) gave themselves some type of sleeping medication to help them go back to sleep.

On the night of the NSNHE, 59.8% (n = 1261) of the respondents indicated having a bed partner who was woken up either intentionally (12.4%, n = 156) or unintentionally (39.7%, n = 501) due to the NSNHE. This suggests that these NSNHEs impact not only the person having the event but also those they sleep with (Table 3).

**Table 3:** Impact on sleep of an NSNHE

	Total	UK	Germany	USA	Canada	France	Italy	Spain	The Netherlands	Sweden	p-Value
Sleep quality/quantity											
How much did this hypo impact on how well you slept, * Mean (s.d.)	5.1 (2.8)	5.6 (2.8)	4.9 (2.7)	5.2 (3.0)	5.2 (2.9)	5.4 (2.9)	4.5 (2.9)	5.1 (2.8)	5.3 (2.4)	4.3 (3.0)	0.000†
From the time the hypo woke you up, how long did it take you to go back to sleep (minutes), Mean (s.d.)	62.6 (61.2)	63.9 (62.0)	56.2 (56.4)	65.4 (62.4)	58.0 (53.2)	56.2 (60.1)	66.5 (57.8)	75.3 (70.9)	60.9 (63.6)	45.1 (46.7)	0.003†
How easy/difficult was it for you to get back to sleep after this hypo, * Mean (s.d.)	5.0 (3.0)	4.9 (2.9)	5.0 (3.0)	5.1 (3.1)	4.9 (3.0)	5.2 (3.1)	5.0 (2.9)	5.1 (2.9)	4.7 (2.7)	3.6 (3.1)	0.025†
Did you take anything (for example, sleeping pills) to go back to sleep? - Yes, N (%)	81 (3.8)	12 (3.9)	5 (1.8)	20 (4.0)	5 (2.5)	10 (5.2)	3 (2.2)	21 (8.7)	2 (1.2)	3 (3.7)	0.002‡
Bed partner											
On the night of the hypo, did you have a bed partner? - Yes, N (%)	1261 (59.8)	187 (61.3)	167 (59.8)	253 (50.4)	105 (52.5)	123 (63.7)	97 (70.2)	155 (64.0)	121 (71.5)	53 (65.4)	0.000‡
On the night of the hypo, did you make an effort *not* to awaken others? - Yes, N (%)	974 (77.2)	160 (85.6)	106 (63.5)	209 (82.6)	83 (79.0)	99 (80.5)	76 (78.4)	119 (76.8)	92 (76.0)	30 (56.6)	0.011‡
On the night of the hypo, did you wake others up on purpose so that they could help you? - Yes, N (%)	156 (12.4)	15 (8.0)	24 (14.4)	38 (15.0)	14 (13.3)	18 (14.6)	8 (8.2)	23 (14.8)	12 (9.9)	4 (7.5)	0.357‡
On the night of the hypo, did you unintentionally wake others (not on purpose)? - Yes, N (%)	501 (39.7)	81 (43.3)	66 (39.5)	99 (39.1)	37 (35.2)	39 (31.7)	54 (55.7)	77 (49.7)	37 (30.6)	11 (20.8)	0.000‡

* 0 to 10-point scale (with 10 being greatest impact).

† Analysis of variance (anova).

‡ Chi square.

#### Impact on Next Day Functioning

It was reported by 79.3% (n = 1672) of respondents that the NSNHE impacted their overall functioning the following day: 39.6% (n = 834) reported feeling emotionally low, 21.4% (n = 452) reported that they avoided driving or drove less, 45.7% (n = 963) found it difficult to concentrate and 46.2% (n = 973) restricted their household chores or errands as well as 27.5% (n = 580) restricting social activities (Table 4). The next day, impact of previous night's poor sleep was evident in that 70.4% reported being tired or fatigued, 60.3% (n = 1273) of respondents reported wanting to take a nap or rest the following day (65.5% of the 1273 did take a nap) and 40.2% (n = 848) reported wanting to go to bed earlier than usual the following night (72.3% of the 848 did go to bed earlier).

**Table 4:** Impact of an NSNHE on next day functioning

	Total	UK	Germany	USA	Canada	France	Italy	Spain	The Netherlands	Sweden	p-Value
Next day functioning											
How much did this hypo impact how you functioned the next day, * Mean (s.d.)	3.9 (3.1)	4.5 (3.1)	4.0 (3.0)	3.9 (3.2)	4.1 (3.0)	4.0 (3.1)	3.0 (3.1)	4.2 (2.9)	3.9 (2.8)	2.1 (2.6)	0.000†
How much did this hypo impact how you felt emotionally the next day, * Mean (s.d.)	4.2 (3.1)	4.5 (3.1)	4.3 (3.0)	4.3 (3.2)	4.3 (3.1)	3.9 (3.1)	3.8 (3.1)	4.3 (2.9)	4.5 (2.8)	2.7 (2.7)	0.000†
The next day, did you want to take a nap and/or rest to catch up on sleep because of your hypo? -Yes, N (%)	1273 (60.3)	214 (70.1)	136 (48.7)	353 (70.4)	133 (66.5)	110 (56.9)	66 (47.8)	124 (51.2)	103 (60.9)	34 (41.9)	0.000‡
The next day, did you take a nap and/or rest? -Yes, N (%)	834 (65.5)	128 (59.8)	96 (70.5)	231 (65.4)	84 (63.1)	68 (61.8)	40 (60.6)	101 (81.4)	65 (63.1)	21 (61.7)	0.008‡
The next day, did you want to go to bed at an earlier time than usual because of your hypo? - Yes, N (%)	848 (40.2)	161 (52.7)	100 (35.8)	209 (41.7)	85 (42.5)	79 (40.9)	47 (34.0)	87 (35.9)	59 (34.9)	21 (25.9)	0.000‡
The next day, did you go to bed earlier? -Yes, N (%)	616 (72.6)	118 (73.2)	70 (70)	140 (66.9)	59 (69.4)	60 (75.9)	36 (76.5)	72 (82.7)	47 (79.6)	14 (66.6)	0.184‡
Restricting social events											
How much did your night time hypo impact your social life,* Mean (s.d.)	2.8 (3.0)	3.1 (3.2)	2.8 (2.9)	2.5 (3.0)	2.7 (2.9)	3.2 (3.1)	2.4 (2.8)	3.1 (2.8)	3.0 (3.0)	1.8 (2.5)	0.001†
Because of the hypo, did you cancel social events OR restrict or limit where you went or what you did for fun? -Yes, N (%)	580 (27.5)	103 (33.7)	87 (31.1)	143 (28.5)	61 (30.5)	62 (32.1)	17 (12.3)	61 (25.2)	33 (19.5)	13 (16.0)	0.000‡
The next day, did you limit your household chores or run fewer errands? -Yes, N (%)	973 (46.1)	153 (50.1)	136 (48.7)	253 (50.4)	99 (49.5)	73 (37.8)	63 (45.6)	105 (43.3)	68 (40.2)	23 (28.3)	0.001‡

* 0 to 10-point scale (with 10 being greatest impact).

† Analysis of variance (anova).

‡ Chi square.

**Figure 3:**
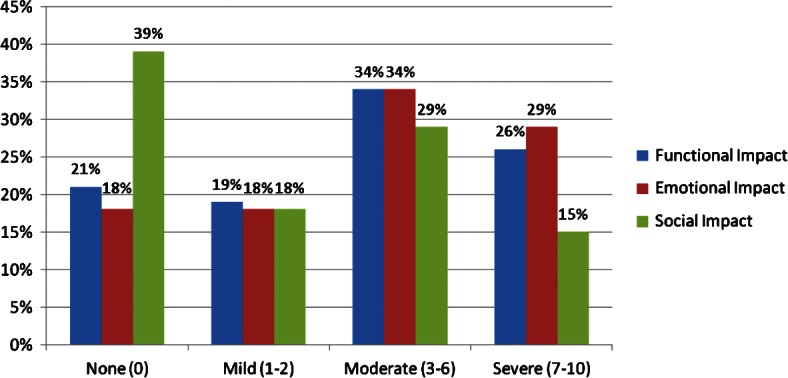
Functional, emotional and social impact on day after NSNHE.

When asked how much their lives were impacted the day after their NSNHEs, 60.7% reported moderate to severe impact on next day functioning, 63.7% for emotional functioning and 43.7% for social functioning (figure 3).

### Comparison with Daytime Hypos

When respondents were asked to compare night-time events to daytime events, night-time events were thought to be more significantly difficult to manage (24.4 vs. 9.4%, p < 0.0001), more severe (25.0 vs. 11.1%, p < 0.0001) and more upsetting (32.3 vs. 13.0%, p < 0.0001). Night-time events also had a significantly greater impact physically (28.5 vs. 12.9%, p < 0.0001), and on the way respondents functioned (25.0 vs. 13.9%, p < 0.0001). In contrast, they found daytime events significantly more frequent than night-time events (35.8 vs. 19.0%, p < 0.001) and about equally as frightening (51.4%).

## Discussion

This study has confirmed that NSNHEs are not uncommon in both T1DM and T2DM patients with diabetes and occur in the past month in approximately 10.4% of patients which is similar although slightly lower than previously found (16.1%) 5. Furthermore, the frequency of events for those who experience NSNHEs is similar, with greater than 50% for both types (61.6% T1DM, 54.2% T2DM) experiencing events at least several times a month. This evidence should further dispel the myth that non-severe events are only a concern to T1DM patients.

Non-severe hypoglycaemia is often considered to be a short-term event, easily dealt with by eating or drinking something that quickly increases blood glucose levels. This study also disproves this clinical myth that these events are ‘minor’ or ‘non-severe’, as these findings show and confirm previous findings 5,6 that these events are not of a short duration nor are they easily managed. According to our findings, there are two distinct phases of hypoglycaemic events: the acute phase (comprised of event recognition and corrective action) and the recovery phase (time needed to return to usual functioning and well-being). It is only the acute phase of the event that is of relatively short duration and generally easily managed by patients. However, the recovery phase is anything but short or easily managed and has clear and highly problematic consequences for patient functioning and well-being not just the night of but also the following day. Of note is the impact on sleep. Sleep is considered to be a necessary prerequisite to optimal functioning and sleep deprivation can lead to increased body mass index and obesity 15, greater prevalence of diabetes or impaired glucose tolerance 16, higher blood pressure or higher prevalence of hypertension 17,18 or cardiovascular disease 19,20. This study shows that NSNHEs have a major impact on both sleep quantity and quality and in fact, 10.4% of patients experiencing an NSNHE remain awake for the remainder of the night and 70.4% feel tired or fatigued the next day. Given the major impacts of NSNHEs identified in previous research as well as this study, calling these events ‘minor’ or ‘non-severe’ is believed to be a serious misnomer and results in a lack of attention both by clinicians and patients to these events. We suggest that calling these events ‘self-treated hypoglycaemia’ would be more accurate as well as less dismissive of their consequences and the recovery phase of the event.

This survey also identified two previously unrecognized serious impacts of NSNHEs, namely falls or injuries due to the event and the impact of NSNHEs on bed partners. Falls and subsequent visits to health care providers are both frightening to patients and represent an additional health care cost. Thus falls and injuries incurred as the result of an NSNHE should be directly addressed by clinicians with patients so that safer strategies for corrective actions can be instituted. This may also be of particular importance to elderly patients as falls in this population represent a major risk factor for increased morbidity and mortality 21. The negative impact of NSNHEs on bed partners suggests that these events are also not inconsequential to others in the household as well as the person with the diabetes and may also impact bed partners next day functioning and well-being. Further research is needed to better understand the prevalence and implications for both of these newly identified consequences of NSNHEs.

NSNHEs also appear to have implications for diabetes management and the role that NSNHEs play in the disruption of optimal glycaemic control should be a considerable clinical concern given that 15.8% of respondents decreased their insulin dose after the NSNHE. Furthermore, the average decrease continued for 3.6 (±5.9) days. When this decrease is multiplied by the high frequency that some patients report having NSNHEs (as often as several times a week), an almost constant, on-going interruption in insulin may result in subset of patients which can create a major barrier to achieving optimal long-term glycaemic control. Further studies are warranted to better understand this link between NSNHEs and glycaemic control.

Country differences, as expected, were found among the nine countries. The sample in Sweden seems to be least impacted by NSNHEs while respondents from the UK expressed greater levels of impact particularly with regards to next-day functioning and sleep.

Several limitations with this study should be mentioned. First, accuracy of reporting, as with any survey, is a consideration as recall bias may have influenced findings. However, recall of episodes of NSHEs up to a week can be considered relatively accurate 11 and recall of longer durations was considered to be accurate as reported by focus group participants in the groups conducted to generate items for this survey. The recall period for most of the sample (76.4%) was within the last 2 weeks and no recall period was longer than 1 month. The fact that this study collected data via an Internet-based survey may also introduce a selection bias in the respondents who were able to participate (i.e. only literate respondents with access to a computer). However, the proportion of Internet users in all nine countries is high (highest in Sweden, 92.2%, to lowest in Italy, 47.7%). Moreover, the rate of literacy is high in all nine countries (99% in most countries with lowest in Spain, 97.7%) 22. Second, accuracy may be impacted by any incentives given the respondents for completion of the survey. Although in this case the amount of the incentive was minimal (approximately US $3–5 depending on country) and should not have affected respondents' decisions to participate in the study. Furthermore, all countries who participated in the study were North American or Western European countries where the similarities in diabetes care can be considered to outweigh the differences. It is unclear if, in countries with more distinct medical systems, cultures or diabetes management pathways, a similar study would yield the same results. Finally, given the panel nature of the survey it was not possible to have a physician confirmed diagnosis. However, it was not known to the patients who completed the screener beforehand that only those with diabetes would be administered the survey. In the screener, the subjects were provided with several medical conditions and asked to check which they had been diagnosed with by a physician. Only those who checked diabetes, among the multiple possibilities, were invited to complete the full survey. It is possible that some patients did misrepresent their diagnosis; however, it is unlikely that this group was large enough to influence findings.

In conclusion, this study strongly suggests that NSNHEs are significant events for patients and negatively impact optimal diabetes management. These events should not be considered ‘minor’ or ‘non severe’ and discussion of these events and optimal corrective action strategies should be incorporated into all diabetes management treatment plans.
